# Joint line restoration during revision total knee arthroplasty: an accurate and reliable method

**DOI:** 10.1186/s40064-015-1543-0

**Published:** 2015-11-26

**Authors:** Chantal Sadaka, Ziad Kabalan, Fadi Hoyek, Georges Abi Fares, Jean-Claude Lahoud

**Affiliations:** Holy Spirit University of Kaslik, Jounieh, Lebanon

**Keywords:** Arthroplasty, Knee, Revision prosthesis, Joint line, Adductor tubercle

## Abstract

During revision total knee arthroplasty, the joint line is frequently malpositioned, due to the disappearance of the anatomical landmarks following previous interventions. This leads to decreased clinical outcome and increased risk of re-intervention. Many methods have been proposed to restore the joint line, but none of them has shown itself to be reliable. We describe an accurate and precise method to localize the exact position of the joint line which guarantees a better clinical knee score. The adductor tubercle (AT) is recognized to be the most reliable landmark used to localize the knee joint line (JL). The distance from the AT to the JL on antero-posterior radiographs (ATJL) and the femoral diameter (FD) on true lateral views were measured on 200 randomly selected normal knees. These measurements were tested for intra- and inter-observer differences. Then, the relationship between these two measurements was studied. A significant correlation and linear regression between FD and ATJL was found (*p* < 0.001), making the adductor tubercle a valid landmark to accurately position the prosthetic joint within 4 mm from the normal position. No significant difference was noted in the intra and inter-observer measurements (F test not significant). Sex was found to be an intervening variable (*p* ˂ 0.001). The correlation and regression between ATJL and FD had to be adjusted accordingly. Once the ATJL was determined preoperatively, the JL level is found during surgery by using a caliper that is held on the easily palpable AT. Knowing the femoral diameter, we can easily locate the joint line level surgically, using the adductor tubercle as a landmark. This method leads to better clinical outcomes and a reduced risk of re-intervention following revision total knee arthroplasty.

## Background

The prevalence of total knee arthroplasty (TKA) is increasing exponentially with a more demanding aging population; this leads to an increase of the number of revision total knee arthroplasty (RTKA) (Popa et al. 2014; James and Bono 2005). In fact, in the USA, between the years 1990 and 2000, the prevalence of TKA performed has increased from 138,552 to 308,250, while the prevalence of RTKA increased from 11,369 to 26,926 (James and Bono 2005). In the year 2011, the number of RTKA performed in the USA (70,000) exceeded by far the number predicted in 2005 for the year 2030 (41,432) (Popa et al. 2014; James and Bono 2005). This remarkable increase in the prevalence of RTKA stresses the need to adjust the operative planning in order to achieve a better clinical outcome.

A good knee score is directly related to the position of the joint line (Yoshii et al. 1991). The latter is often malpositioned with a more frequent tendency to elevation using the available surgical techniques, especially the ones based on balancing the flexion and extension gaps (Partington et al. 1999; Laskin 2002; Romero et al. 2010), as well as the credos that some surgeons rely on: “two finger breadths above the tibial tubercle”; “at the level of the patellar tip on an extended knee” or “2 cm above the fibular head” (Mason et al. 2006). These commonly used methods lack accuracy in positioning the joint line within the narrow acceptable limits of ±8 mm (Figgie et al. 1986; Partington et al. 1999; Laskin 2002), or even ±4 mm (Hofmann et al. 2006) of the optimal position.

After the disappearance of the anatomical landmarks used for the restoration of the joint line, and knowing the deleterious clinical effect of its malposition, we describe a method that is accurate and reproducible based on the adductor tubercle, the distal femoral landmark that can still be intact after the previous procedures and that is the most reliable landmark for this purpose (Iacono et al. 2013).

## Results and discussion

In order to accurately determine the joint line position using plain radiographs of the prosthetic knee to be revised, we considered two radiographic measurements: ATJL and FD.

The one-way ANOVA test did not reveal a significant difference (F test not significant) in the intra and inter-observer reliability of the ATJL and FD measurements (Table 1). These findings are identical to Clement et al. (2014) who also proved the reliability of the FD measurement, as well as Iacono et al. (2013) and Maderbacher et al. Maderbacher et al. 2014) who confirmed likewise the reliability of the ATJL measurement. In addition, Romero et al. (2010) had similar findings concerning distal femoral measurements.

**Table 1:** Results of the one-way ANOVA test for the reliability of the measurements: F values and p values of the one-way ANOVA test, done on the 40 female and 40 male knee measurements (ATJL and FD) taken by the first observer twice and by the two other observers, in order to test for the intra- and inter-observer reliability of these measurements

Measurements tested for intra- and inter-observer differences	F_(df=3;36)_	p value
Female ATJL	0.02	0.997
Male ATJL	0.03	0.992
Female FD	0.07	0.974
Male FD	0.19	0.900

**Fig. 1:**
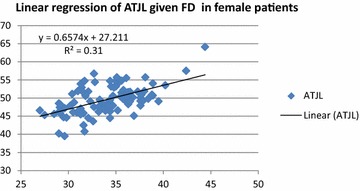
Graph showing the linear regression of ATJL in function of FD in female patients: ATJL = 0.657 FD + 27.211. The coefficient of determination R^2^ is also mentioned

**Fig. 2:**
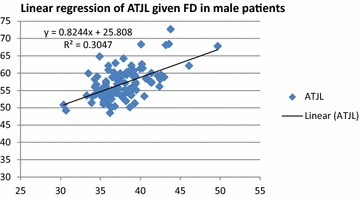
Graph showing the linear regression of ATJL in function of FD in male patients: ATJL = 0.824 FD + 25.808. The coefficient of determination R^2^ is also mentioned

Sex was found to be highly related to ATJL [F_(df=1;198)_ = 158.89; p < 0.001] and to FD [F_(df=1;198)_ = 78.15; p < 0.001]. Therefore, after stratifying by sex as a confounding variable, the following strong correlations and regressions were found between ATJL and FD: For female patients (Fig. 1): ATJL = 0.66 FD + 27.21 [F_(df=1;98)_ = 44.03; p < 0.001]; and for men (Fig. 2): ATJL = 0.82 FD + 25.81 [F_(df=1;98)_ = 42.95; p < 0.001].

Using these formulas, the difference between expected and observed values was limited to 4 mm in 78 % of women and 74 % of men, and to 8 mm in 99 % of women and 97 % of men, proving therefore the high precision of our method in localizing the JL within the narrow acceptable limits of the literature (Figgie et al. 1986; Partington et al. 1999; Laskin 2002). In fact, a cadaveric study has shown that the restoration of the joint line after total knee replacement ensures the normal function of the knee (Yoshii et al. 1991). In addition, the restoration of the joint line guarantees symmetrical flexion and extension gaps, which in turn, warrants knee stability (Hofmann et al. 2006). Figgie et al., based on the modified Mayo Clinic Knee score, as well as Partington et al. who used the Knee Society Score (KSS), demonstrated that an elevation of the joint line of more than 8 mm from its optimal position yielded deleterious clinical outcomes (Figgie et al. 1986; Partington et al. 1999). A newer study showed that the acceptable deviation of the joint line from its normal position is restricted to the interval of 4 mm of elevation or depression, in order to obtain the optimal clinical results (Laskin 2002).

**Fig. 3:**
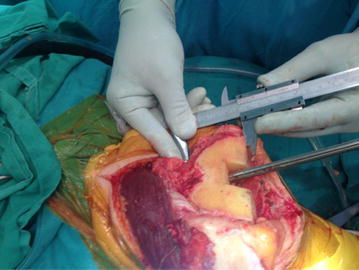
Per-operative ATJL determination: per-operative determination of the adductor tubercle during a revision total knee arthroplasty, and the measurement taken from this landmark to the distal femoral cut. The corresponding measurement will be subtracted from the calculated ATJL, in order to find the corresponding thickness of the prosthesis and augments to be inserted

Once the ATJL is determined preoperatively, the magnification error needs correction. This can be simply done by using a radiology ruler. It can also be done by calculating the magnification ratio between the radiologic and the intra-operative measurement of a segment of the prosthesis to be removed. When the real ATJL distance is known, a caliper can be easily placed on the adductor tubercle and the joint line level will be determined so that the appropriate augment sizes can be selected to restore the bone loss (Fig. 3).

## Conclusions

The restoration of the joint line level during a revision total knee arthroplasty has a major positive effect on the clinical outcome, which leads to reduced risks of re-intervention and precocious complications. No described method showed itself reliable enough to become a standard. The method we described proved itself to be accurate, reproducible, reliable and easily applicable for planning a successful revision total knee arthroplasty.

## Methods

The study design is a level III therapeutic study. The sample was selected from our institution’s database Picture Archiving and Communication System (PACS) at Notre Dame de Secours University Hospital and included 200 knee radiographs that fulfill the following inclusion criteria:

- 100 radiographs of each gender
- Normal knee
- True lateral view
- Good antero-posterior (AP) view
- Age between 20 and 50 years.

Meniscal and ligamentous disorders were not considered as exclusion criteria.

**Fig. 4:**
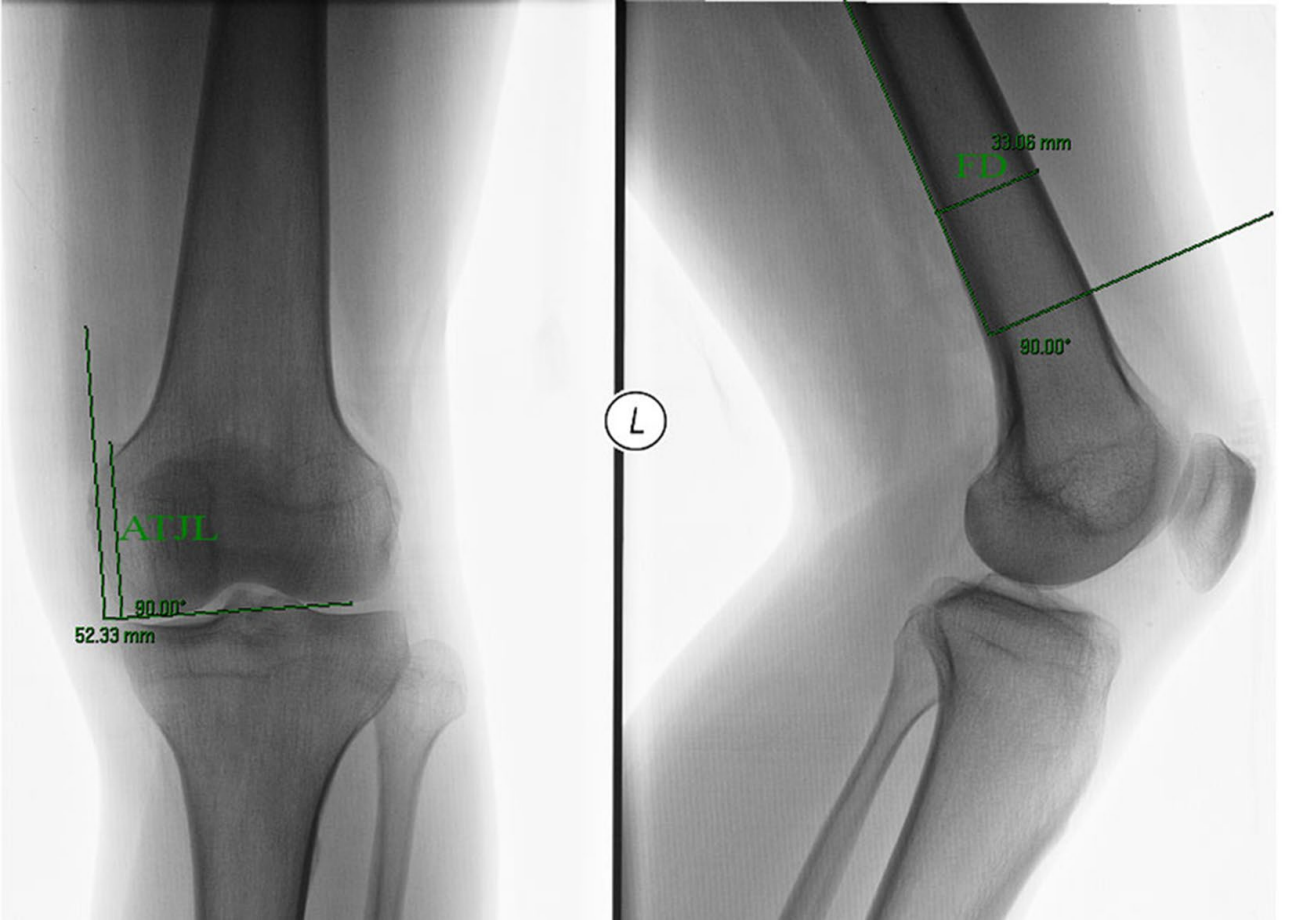
The two radiologic measurements: on the AP view, the distance ATJL from the adductor tubercle to the joint line was measured. On the lateral view, the femoral diameter FD was measured at the level of the flare of the posterior condyle

Two measurements were done on each knee:

1. On the AP view we measured the distance from the adductor tubercle to the joint line, ATJL.
2. On the lateral view the femoral diameter was measured at the level of the flare of the posterior condyle, FD (Fig. 4).

The adductor tubercle was identified on the AP knee radiographs as the most prominent bony protuberance at the summit of the medial condyle.

A first observer, an orthopedic surgeon took the measurements on the 200 radiographs and then repeated them after 2 weeks for 40 male knees and 40 female knees in order to test the method for intra-observer difference. In order to account for inter-observer differences, a radiology resident took measurements on the same 40 male and 40 female knees as a second observer and investigator involved in the making of the study. Then, these same measurements were also taken by a third person, a last year medical student that is completely blind to the study.

The one-way ANOVA test was employed to study the reliability of the intra and inter-observer measurements of FD and ATJL for each gender (F_critical (df=3; 36)_ = 2.87; α = 0.05).

Sex was tested for its effect as an intervening variable. Two simple linear regressions were drawn between sex and FD and between sex and ATJL, in order to determine the presence of a statistically significant relationship between sex and these two variables (F_critical (df =1; 198)_ = 3.89; α = 0.05). After controlling for gender variation, the two measurements done for each knee were studied for correlation, followed by determining the linear regression accordingly (F_critical (df =1; 98)_ = 3.94; α = 0.05).

Intra-operatively, the adductor tubercle serves as a landmark. It is determined as the most prominent bony protuberance on the medial aspect of the distal femur. It can also be palpated at the insertion of the adductor magnus muscle.

## Abbreviations

AT: adductor tubercle
JL: joint line
ATJL: the distance from the Adductor Tubercle to the Joint Line
FD: the femoral diameter measured on the lateral view at the level of the flare of the posterior condyle
TKA: total knee arthroplasty
RTKA: revision total knee arthroplasty
KSS: Knee Society Score
PACS: picture archiving and communication system
AP: antero-posterior
